# Risk of Incident Colorectal Carcinoma in Patients With Type 2 Diabetes on SGLT‐2 Inhibitor Users

**DOI:** 10.1002/pds.70348

**Published:** 2026-03-12

**Authors:** Tsung‐Kun Lin, Wei‐Yao Wang, Jing‐Yang Huang, Yeu‐Sheng Tyan, Mei‐Chun Chen, Gwo‐Ping Jong, Dorji Harnod

**Affiliations:** ^1^ Department of Pharmacy Tri‐Service General Hospital Taipei Taiwan, ROC; ^2^ School of Pharmacy, National Defense Medical University Taipei Taiwan, ROC; ^3^ Department of Internal Medicine Chung Shan Medical University Hospital Taichung Taiwan, ROC; ^4^ School of Medicine, Chung Shan Medical University Taichung Taiwan, ROC; ^5^ Department of Medical Research Chung Shan Medical University Hospital Taichung Taiwan, ROC; ^6^ Department of Medical Imaging Chung Shan Medical University Hospital Taichung Taiwan, ROC; ^7^ Department of Pharmacy Taoyuan Armed Forces General Hospital Taoyuan Taiwan, ROC; ^8^ Department of Emergency and Critical Care Medicine Taipei Medical University SHH Hospital New Taipei City Taiwan, ROC

**Keywords:** cohort study, colorectal carcinoma, SGLT2 inhibitors, type 2 diabetes

## Abstract

**Background:**

The role of sodium‐glucose co‐transporter‐2 inhibitor (SGLT2I) in colorectal carcinoma (CRC) risk in patients with type 2 diabetes mellitus (T2D) remains controversial. This study aimed to examine the association between SGLT2I use and the risk of incident CRC in patients with T2D.

**Methods:**

This nationwide retrospective cohort study was conducted using the National Health Insurance Research Database (2015–2021). The primary outcome was the risk of incident CRC, estimated using hazard ratios (HRs) and 95% confidence intervals (CIs). Multiple Cox regression modeling was conducted to analyze the association between SGLT2I use and incident CRC risk in patients with T2D.

**Results:**

A total of 304 698 SGLT2I users and 609 396 nonusers were matched in a 1:2 ratio by age, sex, and index year from 2 617 996 patients with T2D. Among patients with T2D, 1436 and 3555 incident CRCs were recorded in SGLT2I users and nonusers, respectively. After adjusting for the index year, sex, age, and comorbidities, a significantly decreased risk of CRC was observed among SGLT2I users compared to nonusers (adjusted HR 0.80, 95% CI 0.74–0.85). The sensitivity test for the propensity score 1:1‐matched analyses also showed similar results (adjusted HR 0.79, 95% CI 0.74–0.85).

**Conclusions:**

This population‐based cohort study found that SGLT2I use was associated with a lower risk of CRC than nonuse of SGLT2I in patients with T2D. More studies are needed to evaluate the effects of SGLT2I therapy on CRC prevention in patients with T2D.

## Key Points

- The metabolic dysregulation that occurs in diabetes mellitus (DM) may contribute to colorectal carcinoma (CRC) carcinogenesis and proliferation through inflammation and oxidative stress‐driven signaling alterations.
- Recently, in vitro and in vivo studies have found that sodium‐glucose co‐transporter‐2 inhibitors (SGLT2Is) may reduce CRC risk.
- This study aimed to examine the association between SGLT2I use and the risk of incident CRC in patients with type 2 DM.
- SGLT2I use in patients with type 2 DM decreased the risk of CRC. Moreover, lower risks were also observed in both sexes and age groups among SGLT2I users.
- This study suggests that pharmacological interventions may be beneficial in decreasing the risk of CRC in SGLT2I users.

## Plain Language Summary

- Why was this study conducted?
  ○ Previous studies have shown that diabetes mellitus is associated with an increased risk of colorectal carcinoma. Recent in vitro and in vivo studies have found that sodium‐glucose co‐transporter‐2 inhibitors (SGLT2I) may reduce the colorectal carcinoma (CRC) risk. We performed a population‐based cohort study using Taiwan's National Health Insurance Research Database from 2014 to 2021. We aimed to examine the association between SGLT2I use and the risk of incident CRC in patients with type 2 diabetes (T2D).
- What are the key findings?
  ○ SGLT2I use was associated with a lower risk of CRC than non‐use of SGLT2I in patients with T2D. The lower risks of CRC were also seen in both sexes and age groups in SGLT2I users.
- What does this study add to what is already known?
  ○ Pharmacological interventions may be beneficial in decreasing the risk of CRC in SGLT2I users.

## 1 Introduction

Globally, colorectal cancer (CRC) is the third most common cancer and the second leading cause of cancer‐related mortality from 2018 to 2023 [1]. Specifically, the incidence of early‐onset CRC (age < 50 years) has steadily increased over the past 30 years [2], resulting in a significant health burden [3, 4]. Previous studies have shown that diabetes mellitus (DM) is associated with an increased risk of CRC [5]. The patients with DM and CRC have worse all‐cause mortality and disease‐free survival than CRC patients without DM [6]. Furthermore, the comorbidity of DM with CRC may lead to higher mortality and poorer outcomes. Epidemiological studies also suggest that individuals with diabetes diagnoses may be at increased risk for colorectal cancer (CRC) [7, 8]. The metabolic dysregulation that occurs in DM may contribute to CRC carcinogenesis and proliferation through inflammation and oxidative stress‐driven signaling alterations [7, 8]. Thus, methods to decrease CRC incidence in patients with DM are urgently needed.

Sodium‐glucose co‐transporter‐2 inhibitors (SGLT2Is) are the latest class of antidiabetic and heart failure medications that target the SGLT2 protein and inhibit glucose absorption from the proximal tubule of the kidney, thereby reducing blood sugar levels [9, 10, 11]. Recently, in vitro and in vivo studies have found that SGLT2Is may reduce CRC risk. SGLT2Is have demonstrated anticancer effects in various cancer models through different mechanisms, such as effects on mitochondrial membrane instability, increased cell cycle arrest and apoptosis, suppression of β‐catenin and PI3K‐Akt pathways, and downregulation of oxidative phosphorylation [12, 13, 14]. They also reduce glucose uptake and inhibit tumor growth by their mechanism of blockage of reabsorption of glucose in the renal proximal tubule, hindering glucose absorption for the growth of tumor cells [14, 15]. These findings highlight the need to investigate the association between SGLT2Is and CRC in patients with DM. However, only one epidemiological study has explored this possibility. According to Sung et al., SGLT2I users had a significantly lower risk of developing CRC than dipeptidyl peptidase 4 inhibitor users [16]. Nevertheless, their study involved a comparison of two drug classes rather than a comparison between drug users and nonusers.

Considering the above reports, we test this hypothesis and we conducted a population‐based study aimed to investigate the occurrence of colorectal cancer among SGLT2I users compared to SGLT2I nonusers in patients with type 2 diabetes (T2D) using data from the Health and Welfare Data Science Center (HWDC).

## 2 Materials and Methods

### 2.1 Study Design

This retrospective population‐based cohort study used data from Taiwan's National Health Insurance Research Database (NHIRD) of the HWDC from 2014 to 2021. The NHIRD contains healthcare information for > 23 million people, with a > 99% coverage rate of Taiwanese residents, and has been validated as a robust research database [17, 18, 19]. This study also adhered to the Strengthening the Reporting of Observational Studies in Epidemiology reporting guidelines.

### 2.2 Participants and Recruitment

This study included adults (aged ≥ 20 years) with T2D [International Classification of Diseases, 10th Revision, Clinical Modification (ICD‐10‐CM) code E11], who were treated with the maximum tolerated labeled dose of an SGLT2I on admission to the hospital or as outpatients, between May 2016 and December 2020.

SGLT2I users were patients who received at least one SGLT2I prescription for 180 days during the study period. Non‐SGLT2I users were randomly selected patients with T2D who did not receive any SGLT2I prescriptions throughout the study period. The inclusion criteria were as follows: (1) had two or more outpatient visits within 12 months, (2) continuously received antidiabetic medications for > 6 months during the study period, or (3) had one or more admissions with a diagnosis of T2D. Comorbidities related to CRC were recorded according to the ICD‐10‐CM code and included chronic kidney disease (ICD‐10‐CM code N18), hypertension (ICD‐10‐CM code I10), hyperlipidemia (ICD‐9‐CM code E78.1–E78.5), chronic liver disease (ICD‐10‐CM code K71, K75, and K76), chronic obstructive pulmonary disease (ICD‐10‐CM code J44), and peptic ulcer (ICD‐10‐CM code K27). The exclusion criteria between two groups were as follows: (1) a history of CRC or other cancers before the index date, (2) follow‐up of < 6 months, and (3) aged < 20 years. To account for the differences in baseline characteristics and CRC risk between SGLT2I users and non‐users, the groups were matched for age, sex, and DM duration at a ratio of 1:2. The index date was defined as the first SGLT‐2I prescription between May 2016 and December 2020. For the SGLT2i non‐users group, the index date corresponded to that of their matched counterparts in the SGLT2i users group.

### 2.3 Variables and Study Outcomes

All baseline characteristics were assessed on the index date. The baseline variables included sex, age, diabetes duration, comorbidities, and concurrent medication. Comorbidities made within 6 months before the index date and medication use were restricted to prescriptions made within 6 months before the index date. The study endpoint was CRC development, defined as the first occurrence of a CRC code (ICD‐10‐CM codes C18‐C21) in inpatient or outpatient claim records after the index date during follow‐up or follow‐up to December 2021.

### 2.4 Data Analysis

The number, percentage, and standard deviation of patients meeting each baseline characteristic were reported. The descriptive statistics of the two groups are presented as numbers and percentages. Absolute standardized differences (ASD) were calculated to analyze the variable distribution differences between SGLT‐2i users and non‐users. An ASD of ≤ 0.1 was regarded as negligible. In both groups, the incidence rates of CRC were calculated per 10 000 person‐months. The crude hazard ratios (HRs) and 95% confidence intervals (CIs) of incidence rate ACS were estimated by using Cox proportional hazard regression. Multivariable models were further used to calculate the adjusted HRs and 95% CIs for cancer risk after adjustment for sex, age, DM duration, comorbidities, and concomitant medication use. Furthermore, the Kaplan–Meier method was employed to identify differences in the cumulative incidence of CRC between SGLT‐2i users and non‐users.

A sensitivity analysis was also conducted to test the robustness of our primary findings. A propensity score 1:1 matching was performed to balance baseline covariates between the groups. Then, the ASD was calculated to estimate the difference between the two groups. An ASD ≤ 0.10 implies a negligible difference in potential confounders between the two groups. Furthermore, the Kaplan–Meier method was also employed to identify differences in the cumulative incidence of CRC between SGLT‐2i users and non‐users.

Subgroup analyses were performed, stratified by sex and age comorbidity at baseline for the primary outcomes of CRC. Finally, multiple Cox regression to estimate the hazard ratio for comorbidity at baseline were also performed for the primary outcomes of CRC. The results are presented as HRs with 95% CIs. Significance was considered at *p* < 0.05. All statistical calculations were performed using statistical analysis software, version 9.4 (SAS Institute Inc., Cary, NC, USA).

## 3 Results

**FIGURE 1:**
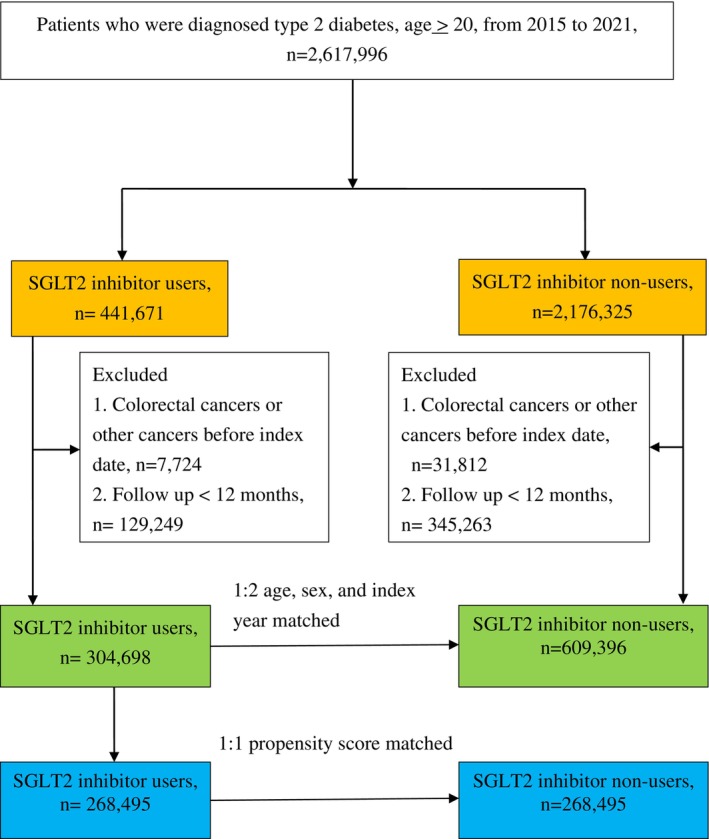
Patient flowchart. SGLT2: sodium‐glucose co‐transporter‐2.

From May 2016 through December 2021, 441 671 type 2 DM patients received their first SGLT2I prescriptions. After exclusions, 304 698 individuals in the study group and 609 396 control patients were matched for sex, age, and index year at a 1:2 ratio (Figure 1). The mean follow‐up period of the study subjects was 3.0 years. SGLT2I users had more comorbidities with hypertension, hyperlipidemia, heart failure, and chronic liver disease at baseline and used more concurrent medications than non‐users (Table 1).

**TABLE 1:** Baseline characteristics.

	2:1 sex, age, and index year matching			After 1:1 PSM		
	Non‐ SGLT2I	SGLT2I	ASD	Non‐ SGLT2I	SGLT2I	ASD
*N*	609 396	304 698		268 495	268 495	
Index year			0.0000			0.0287
2016	22 416 (3.68%)	11 208 (3.68%)		10 226 (3.81%)	9718 (3.62%)	
2017	160 346 (26.31%)	80 173 (26.31%)		71 611 (26.67%)	69 591 (25.92%)	
2018	146 852 (24.10%)	73 426 (24.10%)		64 298 (23.95%)	64 699 (24.10%)	
2019	141 202 (23.17%)	70 601 (23.17%)		61 544 (22.92%)	62 701 (23.35%)	
2020	138 580 (22.74%)	69 290 (22.74%)		60 816 (22.65%)	61 786 (23.01%)	
Sex			0.0000			0.0072
Female	261 402 (42.90%)	130 701 (42.90%)		114 283 (42.56%)	115 234 (42.92%)	
Male	347 994 (57.10%)	173 997 (57.10%)		154 212 (57.44%)	153 261 (57.08%)	
Age			0.0000			0.0000
20–49	127 521 (20.93%)	64 614 (21.21%)		57 459 (21.40%)	56 545 (21.06%)	
50–59	168 542 (27.66%)	83 873 (27.53%)		74 874 (27.89%)	74 484 (27.74%)	
60–69	197 449 (32.40%)	98 402 (32.29%)		86 289 (32.14%)	87 112 (32.44%)	
≥ 70	115 884 (19.02%)	57 809 (18.97%)		49 873 (18.58%)	50 344 (18.75%)	
Mean (SD)	59.22 (12.21)	59.15 (12.25)		59.15 (11.89)	59.10 (12.23)	
Type 2 diabetes history			0.0252			0.0417
≤ 2 years	57 588 (9.45%)	27 026 (8.87%)		24 111 (8.98%)	23 681 (8.82%)	
3–4 years	368 014 (60.39%)	182 088 (59.76%)		163 406 (60.86%)	160 882 (59.92%)	
≥ 5 years	183 794 (30.16%)	95 584 (31.37%)		80 978 (30.16%)	83 932 (31.26%)	
Comorbidities						
Hypertension	290 301 (47.64%)	162 157 (53.22%)	0.1118	140 672 (52.39%)	140 584 (52.36%)	0.0007
Hyperlipidemia	268 725 (44.10%)	169 479 (55.62%)	0.2321	146 644 (54.62%)	146 324 (54.50%)	0.0024
Chronic kidney disease	56 771 (9.32%)	21 216 (6.96%)	0.0861	18 784 (7.00%)	19 007 (7.08%)	0.0033
Chronic liver disease	42 275 (6.94%)	22 931 (7.53%)	0.0227	20 429 (7.61%)	20 101 (7.49%)	0.0046
COPD	14 522 (2.38%)	7068 (2.32%)	0.0042	5966 (2.22%)	6134 (2.28%)	0.0042
Peptic ulcer	172 456 (28.30%)	84 934 (27.87%)	0.0095	74 294 (27.67%)	74 564 (27.77%)	0.0023
Medication						
NSAIDs	419 188 (68.79%)	212 859 (69.86%)	0.0232	186 189 (69.35%)	186 818 (69.58%)	0.0051
Corticosteroids	165 988 (27.24%)	84 278 (27.66%)	0.0094	73 217 (27.27%)	73 524 (27.38%)	0.0026
Aspirin	143 361 (23.53%)	94 084 (30.88%)	0.1658	77 595 (28.90%)	78 263 (29.15%)	0.0055
Statin	344 438 (56.52%)	229 756 (75.40%)	0.4067	196 669 (73.25%)	196 632 (73.23%)	0.0003
Biguanides	444 774 (72.99%)	285 594 (93.73%)	0.5799	250 393 (93.26%)	249 443 (92.90%)	0.0139
Sulfonylureas	194 182 (31.86%)	137 531 (45.14%)	0.2753	117 089 (43.61%)	115 231 (42.92%)	0.0140
Alpha glucosidase inhibitors	59 627 (9.78%)	59 772 (19.62%)	0.2804	43 760 (16.30%)	44 963 (16.75%)	0.0121
Thiazolidinediones	61 551 (10.10%)	63 766 (20.93%)	0.3025	47 483 (17.68%)	48 494 (18.06%)	0.0098
DPP4	135 956 (22.31%)	128 932 (42.31%)	0.4379	99 100 (36.91%)	99 911 (37.21%)	0.0063
Insulin	102 354 (16.80%)	81 315 (26.69%)	0.2415	63 436 (23.63%)	63 683 (23.72%)	0.0022
GLP‐1	8045 (1.32%)	8718 (2.86%)	0.1079	6396 (2.38%)	6562 (2.44%)	0.0040

Abbreviations: ASD: absolute standardized difference, COPD: chronic obstructive pulmonary disease, DPP4: dipeptidyl peptidase 4, GLP‐1: Glucagon‐like peptide‐1, NSAID: non‐steroidal anti‐inflammatory drug, PSM: propensity score matching, SD: Standard Deviation, SGLT2I: sodium‐glucose co‐transporter‐2 inhibitor.

### 3.1 Relative Risk of CRC in Patients Matched for Sex, Age, and Index Year at a 1:2 Ratio

**FIGURE 2:**
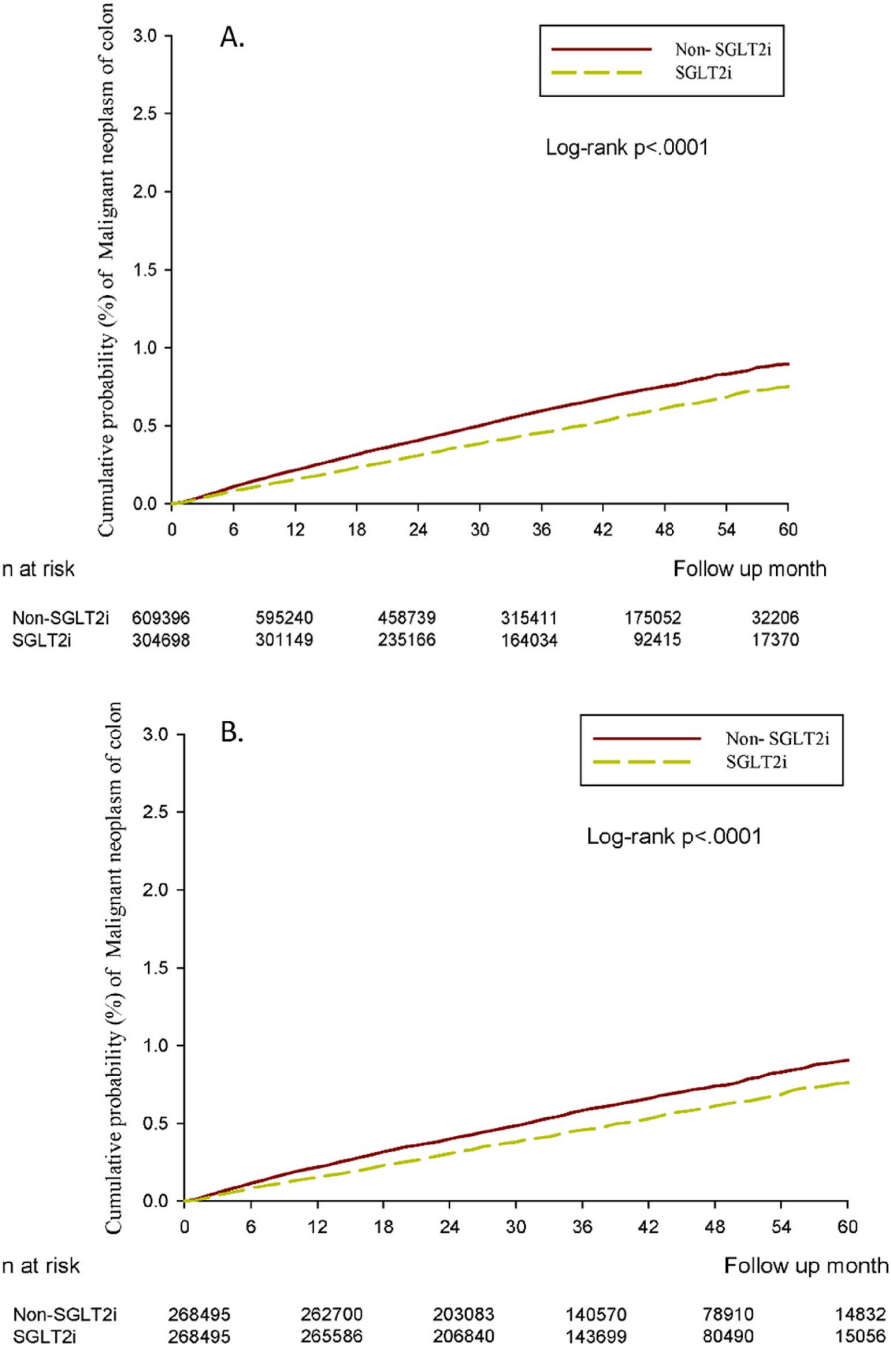
(A) Cumulative risk curve of incidental colorectal carcinoma for the study cohorts treated with SGLT2 inhibitor users versus non‐SGLT2 inhibitor users. (B) Cumulative risk curve of incidental colorectal carcinoma for the study cohorts under propensity score matching treated with SGLT2 inhibitor users versus non‐SGLT2 inhibitor users. SGLT2i: sodium‐glucose co‐transporter‐2 inhibitor.

The crude incidence rate of CRC was 13.02 per 100 000 person‐months (95% CI 12.37–13.72) for SGLT2I users compared with 16.52 (95% CI 15.98–17.07) for non‐SGLT2I users. A significantly lower incidence of CRC was found in SGLT2I users than in non‐users (crude HR, 0.79; 95% CI 0.74–0.84) (Table 2). The results were not substantially changed after adjustments for the index date, sex, age, comorbidities, and concurrent medications at baseline (aHR, 0.80; 95% CI 0.74–0.85). The effects of SGLT2 inhibitors on CRC incidence were demonstrated in a Kaplan–Meier plot (Figure 2A). Moreover, the cumulative incidence of CRC (*p* < 0.0001) was lower in SGLT2I users than in non‐SGLT2I users.

**TABLE 2:** Incidence rate of colorectal carcinoma.

	2:1 sex, age, & index date matching		After 1:1 PSM	
	Non‐SGLT2I users	SGLT2I users	Non‐ SGLT2I users	SGLT2I users
*N*	609 396	304 698	268 495	268 495
Follow up person months	21 523 387	11 025 088	9 545 094	9 687 589
New case	3555	1436	1557	1264
Incidence rate^a^ (95% CI)	16.52 (15.98–17.07)	13.02 (12.37–13.72)	16.31 (15.52–17.14)	13.05 (12.35–13.79)
Crude Relative risk (95% CI)	Reference	0.79 (0.74–0.84)	Reference	0.80 (0.74–0.86)
Adjusted HR (95% CI)^b^	Reference	0.80 (0.74–0.85)	Reference	0.79 (0.74–0.85)

Abbreviations: CI: confidence interval, HR: hazard ratio, PSM: propensity score matching, SGLT2I: sodium‐glucose co‐transporter‐2 inhibitor.

^a^ Incidence rate, per 100 000 person‐months.

^b^ Adjusted hazard ratio, the covariates including year of index, sex, age, co‐morbidities, and medication at baseline.

### 3.2 Sensitivity Analysis of the Relative Risk of RCC in a Propensity Score 1:1 Matching

A sensitivity analysis of the relative risk of CRC in a propensity score 1:1 matching analysis was conducted. Initially, the Cox regression analysis indicated that SGLT2I users had a 20% reduction compared with non‐users (crude HR, 0.80; 95% CI 0.74–0.86) (Table 2). After adjusting the index date, sex, age, comorbidities, and concurrent medication, the results were consistent with the main findings (aHR, 0.79; 95% CI 0.74–0.85; Table 2). In the Kaplan–Meier analysis, a clear separation of event curves for the cumulative incidence of CRC was found between these two groups (Figure 2B).

### 3.3 Subgroup and Comorbidity Analysis

Subgroup analyses were conducted to compare the HRs of the study outcomes between SGLT2I users and non‐SGLT2I users. Similar results were also seen for sex (male and female) and all age groups (Table 3). However, an overall null effect of SGLT2I use on CRC risk was seen for comorbidity with hyperlipidemia, chronic kidney disease, and peptic disease compared with non‐comorbidity with hyperlipidemia, chronic kidney disease, and peptic disease, whereas comorbidity with chronic obstructive pulmonary disease (COPD) significantly increased the CRC risk compared with non‐comorbidity with COPD (Table 4).

**TABLE 3:** Subgroup analysis.

	*N*	Follow up person months	New case	Incidence rate^a^ (95% CI)		HR (95% CI)	aHR^b^ (95% CI)
				Non‐ SGLT2	SGLT2		
Sex							
Female	392 103	14 289 594	1954	14.26 (13.52–15.04)	12.53 (11.57–13.57)	0.88 (0.80–0.97)	0.85 (0.76–0.94)
Male	521 991	18 258 881	3037	18.29 (17.54–19.07)	13.41 (12.53–14.35)	0.74 (0.68–0.80)	0.76 (0.70–0.83)
*p* for interaction							0.0071
Age							
< 50	192 135	7 095 402	401	6.11 (5.45–6.87)	4.76 (3.96–5.71)	0.78 (0.63–0.97)	0.83 (0.65–1.05)
50–59	252 415	9 319 155	1038	12.38 (11.53–13.29)	8.69 (7.72–9.79)	0.70 (0.61–0.81)	0.68 (0.58–0.78)
60–69	295 851	10 573 328	1976	20.27 (19.25–21.36)	15.58 (14.34–16.93)	0.77 (0.70–0.85)	0.80 (0.72–0.89)
≥ 70	173 693	5 560 590	1576	29.59 (27.88–31.40)	25.94 (23.74–28.33)	0.88 (0.79–0.98)	0.87 (0.78–0.98)
*p* for interaction							0.0620
< 60	444 550	16 414 557	1439	9.68 (9.11–10.29)	6.98 (6.32–7.71)	0.72 (0.64–0.81)	0.71 (0.63–0.81)
≥ 60	469 544	16 133 918	3552	23.47 (22.57–24.41)	19.17 (18.05–20.37)	0.82 (0.76–0.88)	0.83 (0.77–0.90)
*p* for interaction							0.0474

Abbreviations: CI: confidence interval, HR: hazard ratio, SGLT2I: sodium‐glucose co‐transporter‐2 inhibitor.

^a^ Incidence rate, per 100 000 person‐months.

^b^ Adjusted hazard ratio, the covariates including year of index, sex, age, co‐morbidities, and medication at baseline.

**TABLE 4:** Multiple Cox regression to estimate the hazard ratio for comorbidity.

Comorbidity (ref: non‐comorbidity)	HR (95% CI)	aHR (95% CI)
Hypertension	1.13 (1.06–1.19)	1.14 (1.05–1.23)
Hyperlipidemia	0.99 (0.93–1.05)	0.98 (0.91–1.06)
Chronic kidney disease	1.17 (1.06–1.30)	1.13 (0.98–1.30)
Chronic liver disease	1.10 (0.99–1.23)	1.12 (0.97–1.28)
COPD	1.23 (1.06–1.43)	1.24 (1.01–1.51)
Peptic ulcer	0.95 (0.88–1.02)	0.95 (0.86–1.05)

Abbreviations: CI: confidence interval, COPD: chronic obstructive pulmonary disease, HR: hazard ratio, SGLT2I: sodium‐glucose co‐transporter‐2 inhibitor.

## 4 Discussion

This population‐based cohort study showed a significantly lower risk of CRC in SGLT2I users than in non‐SGLT2I users. Lower risks were also observed in both sexes and all age groups. However, the risk was lower in women than in men. In this study, comorbidity with COPD significantly increased CRC risk compared with non‐comorbidity with COPD.

The mechanisms by which SGLT2I reduces CRC risk are plausible. In in vivo and in vitro studies, the antineoplastic effect of SGLT2I is thought to be mediated by sirtuin 3 upregulation, apoptotic cell death, cell cycle arrest, autophagy, and endoplasmic reticulum stress in CRC cells [13, 16, 20, 21]. SGLT2I also counteracts proinflammatory phenotypes and high insulin levels [22]. Numerous experimental studies have demonstrated that insulin can induce the proliferation of colorectal epithelial cells and the development of aberrant crypt foci (CRC cells) [23, 24, 25]. Epidemiological studies have shown that patients with high glycated hemoglobin (HbA1c) levels have increased CRC risk [26, 27]. Furthermore, since SGLT2I can better control blood glucose levels, variability, and complications in patients with type 2 DM, it may more effectively prevent CRC [27, 28]. These findings align with the conclusion that antidiabetic medications such as SGLT2I are associated with a reduced risk of incident CRC.

Regarding the preventive effect of SGLT2I on cancer, one possibility is that when prescribing a new drug, doctors may be more attentive and provide more lifestyle improvement advice to enhance patient compliance, which could positively influence prognosis. Therefore, there is a pressing need for large‐scale clinical trials with robust evidence‐based medicine to confirm the preventive effect of SGLT2I on cancer in the future.

Consistent with the results reported in previous literature, our findings showed a significantly lower risk of CRC in sodium‐glucose co‐transporter‐2 inhibitor (SGLT2I) users than in non‐SGLT2I users among patients with T2D [29, 30, 31, 32]. Suzuki et al. evaluated 26 823 patients and found that SGLT2I users are associated with a reduced CRC risk compared to DPP‐4 inhibitors in patients with type 2 diabetes [30]. Simultaneously, a meta‐analysis also demonstrated that SGLT2I users might have a more preventive effect on CRC than DPP‐4 inhibitors [29]. Another study also reported that SGLT2I users were most effective for the prevention of CRC than metformin [32]. Therefore, the preventive effect of CRC among individual SGLT2I users is certain.

Previous epidemiological studies have revealed a clear sex difference in CRC incidence [27, 28, 33]. Generally, women have a lower incidence of CRC than men. This study indicated that SGLT2Is were associated with a significantly reduced CRC risk in both sexes, with the risk being lower among women than men. The reasons for the differences in this association by sex are not fully understood. Variations in dietary factors, tumor characteristics, treatment, sedentary lifestyle, gene mutation rates, and etiological pathways for the effects of SGLT2Is on CRC risk may explain the heightened risk in men, while most risks remain unknown [28, 34]. Further clinical studies are necessary to test the potential effect modification by sex and validate our results.

Interestingly, this study showed that SGLT2i users were significantly associated with decreased colorectal cancer risk with age (≥ 60 vs. < 60 years old) in the subgroup analysis. Age over 50 years is a leading risk factor for CRC [35, 36]. Importantly, CRC diagnosed in patients under the age of 50 years has been increasing around the world in recent years [35]. Our finding can be modified this risk factor. Further randomized clinical trials are required to confirm current study findings in the future.

Previous observational studies have revealed that patients with COPD have an increased risk of incident CRC compared to those without COPD [37, 38]. This study indicated a higher risk of CRC in SGLT2I users with type 2 DM and COPD than in those without COPD. COPD is a chronic inflammatory condition often associated with elevated levels of proinflammatory cytokines [38, 39]. When these cytokines enter the gastrointestinal tract, they may promote alterations in metabolic products [39]. In lung epithelial cells of patients with COPD, disturbances in lipid metabolism products play a critical role in the growth of CRC cells [40]. To the best of our knowledge, this is the first study to investigate the effect of SGLT2I use in patients with type 2 DM and COPD. Further comprehensive in vivo, in vitro, and clinical studies are needed to confirm these findings.

This study had several limitations. First, there was a lack of information regarding patient lifestyle factors, such as smoking, physical activity, alcohol intake, and personal details, following decoding in the NHIRD. Therefore, we could not account for these confounders in our data analyses. However, a similar method for identifying patients between the SGLT2I and non‐SGLT2I groups in the same dataset has been utilized and proven valid in the previously published studies [41, 42, 43]. Second, we utilized ICD‐10‐CM codes to identify diagnoses, outcomes, and comorbidities in the NHIRD, which were registered by each physician and may have been miscoded or misclassified. Third, detailed clinical, laboratory, endoscopic, and radiological data were lacking in the NHIRD. Additionally, the severity of CRC could not be investigated in this study. Therefore, our findings may not be applicable to patients from other countries.

## 5 Conclusions

SGLT2I use in patients with T2D or heart failure decreased the risk of CRC. Moreover, lower risks were also observed in both sexes and age groups among SGLT2I users. Our findings suggest that pharmacological interventions may be beneficial in decreasing the risk of CRC in SGLT2I users. Increased awareness should be promoted among treating physicians regarding this potentially lower risk of CRC in patients with T2D.

## Funding

The study was supported by the grant from Taoyuan Armed Forces General Hospital (TYAFGH‐A‐112004).

## Conflicts of Interest

The authors declare no conflicts of interest.

## Data Availability

The authors have nothing to report.

## References

[1] R. L. Siegel , K. D. Miller , N. S. Wagle , and A. Jemal , “Cancer Statistics, 2023,” CA: a Cancer Journal for Clinicians 73 (2023): 17–48, 10.3322/caac.21763. 36633525

[2] M. R. Saraiva , I. Rosa , and I. Claro , “Early‐Onset Colorectal Cancer: A Review of Current Knowledge,” World Journal of Gastroenterology 29 (2023): 1289–1303, 10.3748/wjg.v29.i8.1289. 36925459 PMC10011966

[3] L. Klimeck , T. Heisser , M. Hoffmeister , and H. Brenner , “Colorectal Cancer: A Health and Economic Problem,” Best Practice & Research Clinical Gastroenterology 66 (2023): 101839, 10.1016/j.bpg.2023.101839. 37852707

[4] S. G. Patel and J. A. Dominitz , “Screening for Colorectal Cancer,” Annals of Internal Medicine 177 (2024): ITC49‐64, 10.7326/AITC202404160. 38588547

[5] T. Lawler , Z. L. Walts , M. Steinwandel , et al., “Type 2 Diabetes and Colorectal Cancer Risk,” JAMA Network Open 6 (2023): e2343333, 10.1001/jamanetworkopen.2023.43333. 37962884 PMC10646729

[6] J. Li , J. Liu , C. Gao , F. Liu , and H. Zhao , “Increased Mortality for Colorectal Cancer Patients With Preexisting Diabetes Mellitus: An Updated Meta‐Analysis,” Oncotarget 8 (2017): 62478–62488, 10.18632/oncotarget.19923. 28977962 PMC5617522

[7] X. Mao , K. S. Cheung , J. T. Tan , et al., “Optimal Glycaemic Control and the Reduced Risk of Colorectal Adenoma and Cancer in Patients With Diabetes: A Population‐Based Cohort Study,” Gut 73 (2024): 1313–1320, 10.1136/gutjnl-2023-331701. 38569845

[8] H. D. Khoa Ta , N. N. Nguyen , D. K. N. Ho , et al., “Association of Diabetes Mellitus With Early‐Onset Colorectal Cancer: A Systematic Review and Meta‐Analysis of 19 Studies Including 10 Million Individuals and 30,000 Events,” Diabetes & Metabolic Syndrome 17 (2023): 102828, 10.1016/j.dsx.2023.102828. 37490785

[9] M. Duran , M. Ziyrek , and Y. Alsancak , “Effects of SGLT2 Inhibitors as an Add‐On Therapy to Metformin on Electrocardiographic Indices of Ventricular Repolarization,” Acta Cardiologica Sinica 36 (2020): 626–632, 10.6515/ACS.202011_36(6).20200511A. 33235419 PMC7677636

[10] A. J. Scheen , “The Current Role of SGLT2 Inhibitors in Type 2 Diabetes and Beyond: A Narrative Review,” Expert Review of Endocrinology and Metabolism 18 (2023): 271–282, 10.1080/17446651.2023.2210673. 37154218

[11] S. Nazari and H. Mirkhani , “Cardiorenal Protections of SGLT2 Inhibitors in the Treatment of Type 2 Diabetes,” Current Diabetes Reviews 19 (2023): e221222212126, 10.2174/1573399819666221222160035. 36567296

[12] M. Dutka , R. Bobiński , T. Francuz , et al., “SGLT‐2 Inhibitors in Cancer Treatment‐Mechanisms of Action and Emerging New Perspectives,” Cancers 14 (2022): 5811, 10.3390/cancers14235811. 36497303 PMC9738342

[13] J. Kato , Y. Shirakami , M. Ohnishi , et al., “Suppressive Effects of the Sodium‐Glucose Cotransporter 2 Inhibitor Tofogliflozin on Colorectal Tumorigenesis in Diabetic and Obese Mice,” Oncology Reports 42 (2019): 2797–2805, 10.3892/or.2019.7357. 31638239

[14] R. Benedetti , G. Benincasa , K. Glass , et al., “Effects of Novel SGLT2 Inhibitors on Cancer Incidence in Hyperglycemic Patients: A Meta‐Analysis of Randomized Clinical Trials,” Pharmacological Research 175 (2022): 106039, 10.1016/j.phrs.2021.106039. 34929299

[15] D. K. McGuire , W. J. Shih , F. Cosentino , et al., “Association of SGLT2 Inhibitors With Cardiovascular and Kidney Outcomes in Patients With Type 2 Diabetes: A Meta‐Analysis,” JAMA Cardiology 6 (2021): 148–158, 10.1001/jamacardio.2020.4511. 33031522 PMC7542529

[16] H. L. Sung , C. Y. Hung , Y. C. Tung , C. C. Lin , T. H. Tsai , and K. H. Huang , “Comparison Between Sodium‐Glucose Cotransporter 2 Inhibitors and Dipeptidyl Peptidase 4 Inhibitors on the Risk of Incident Cancer in Patients With Diabetes Mellitus: A Real‐World Evidence Study,” Diabetes/Metabolism Research and Reviews 40 (2024): e3784, 10.1002/dmrr.3784. 38402457

[17] J. Y. Yang , Y. W. Wu , W. Chuang , et al., “An Integrated Community‐Based Blood Pressure Telemonitoring Program ‐ A Population‐Based Observational Study,” Acta Cardiologica Sinica 38 (2022): 612–622, 10.6515/ACS.202209_38(5).20220330A. 36176366 PMC9479044

[18] C. C. Chang , S. Y. Wu , Y. R. Lai , et al., “The Utilization of Chinese Herbal Products for Hyperthyroidism in National Health Insurance System (NHIRD) of Taiwan: A Population‐Based Study,” Evidence‐based Complementary and Alternative Medicine 2022 (2022): 5500604, 10.1155/2022/5500604. 35449810 PMC9017513

[19] L. G. Huang , C. C. Yu , M. C. Lin , Y. H. Wang , and Y. C. Chang , “Association Between Periodontitis and Hematologic Cancer: An NHIRD Cohort Study in Taiwan,” Cancers 16 (2024): 1671, 10.3390/cancers16091671. 38730623 PMC11083422

[20] C. Anastasio , I. Donisi , V. Del Vecchio , et al., “SGLT2 Inhibitor Promotes Mitochondrial Dysfunction and ER‐Phagy in Colorectal Cancer Cells,” Cellular and Molecular Biology Letters 29 (2024): 80, 10.1186/s11658-024-00599-1. 38811901 PMC11134909

[21] I. V. Madunić , J. Madunić , D. Breljak , D. Karaica , and I. Sabolić , “Sodium‐Glucose Cotransporters: New Targets of Cancer Therapy?,” Arhiv za Higijenu Rada i Toksikologiju 69 (2018): 278–285, 10.2478/aiht-2018-69-3204. 30864374

[22] H. Yan , C. Huang , X. Shen , J. Li , S. Zhou , and W. Li , “GLP‐1 RAs and SGLT‐2 Inhibitors for Insulin Resistance in Nonalcoholic Fatty Liver Disease: Systematic Review and Network Meta‐Analysis,” Frontiers in Endocrinology 13 (2022): 923606, 10.3389/fendo.2022.923606. 35909522 PMC9325993

[23] J. Okada , E. Yamada , T. Saito , et al., “Dapagliflozin Inhibits Cell Adhesion to Collagen I and IV and Increases Ectodomain Proteolytic Cleavage of DDR1 by Increasing ADAM10 Activity,” Molecules 25 (2020): 495, 10.3390/molecules25030495. 31979355 PMC7038111

[24] T. T. Tran , D. Naigamwalla , A. I. Oprescu , et al., “Hyperinsulinemia, but Not Other Factors Associated With Insulin Resistance, Acutely Enhances Colorectal Epithelial Proliferation in Vivo,” Endocrinology 147 (2006): 1830–1837, 10.1210/en.2005-1012. 16410309

[25] C. H. Chiang , C. H. Chiang , Y. P. Hsia , et al., “The Impact of Sodium‐Glucose Cotransporter‐2 Inhibitors on Outcome of Patients With Diabetes Mellitus and Colorectal Cancer,” Journal of Gastroenterology and Hepatology 39 (2024): 902–907, 10.1111/jgh.16498. 38296226

[26] K. T. Khaw , N. Wareham , S. Bingham , R. Luben , A. Welch , and N. Day , “Preliminary Communication: Glycated Hemoglobin, Diabetes, and Incident Colorectal Cancer in Men and Women: A Prospective Analysis From the European Prospective Investigation Into Cancer‐Norfolk Study,” Cancer Epidemiology, Biomarkers & Prevention 13 (2004): 915–919. 15184246

[27] J. Lin , P. M. Ridker , A. Pradhan , et al., “Hemoglobin A1c Concentrations and Risk of Colorectal Cancer in Women,” Cancer Epidemiology, Biomarkers & Prevention 14 (2005): 3010–3012, 10.1158/1055-9965.EPI-05-0533. PMC1351341 16365028

[28] F. N. van Erning , N. E. M. Greidanus , R. H. A. Verhoeven , et al., “Gender Differences in Tumor Characteristics, Treatment and Survival of Colorectal Cancer: A Population‐Based Study,” Cancer Epidemiology 86 (2023): 102441, 10.1016/j.canep.2023.102441. 37633058

[29] H. Hajishah , P. Mazloom , A. Salehi , et al., “Comparative Risk of Cancer Associated With SGLT Inhibitors and DPP‐4 Inhibitors in Patients With Diabetes: A Systematic Review and Meta‐Analysis,” Diabetology and Metabolic Syndrome 17 (2025): 321, 10.1186/s13098-025-01898-z. 40770742 PMC12330067

[30] Y. Suzuki , H. Kaneko , A. Okada , et al., “Association of SGLT2 Inhibitors With Incident Cancer,” Diabetes & Metabolism 50 (2024): 101585, 10.1016/j.diabet.2024.101585. 39455021

[31] R. N. C. Chan , R. N. F. Chan , O. H. I. Chou , G. Tse , and S. Lee , “Lower Risks of Incident Colorectal Cancer in SGLT2i Users Compared to DPP4i Users: A Propensity Score‐Matched Study With Competing Risk Analysis,” European Journal of Internal Medicine 110 (2023): 125–127, 10.1016/j.ejim.2023.01.021. 36732129

[32] X. Mao , K. S. Cheung , J. T. Tan , et al., “Risk of Colorectal Cancer and Cancer‐Related Mortality in Type 2 Diabetes Patients Treated With Metformin, SGLT‐2 Inhibitors, or Their Combination,” Cancer Communications 45 (2025): 880–883, 10.1002/cac2.70028. 40275591 PMC12328085

[33] Y. Kang and H. Son , “Gender Differences in Factors Associated With Colorectal Cancer Screening: A National Cross‐Sectional Study in Korea,” Asia‐Pacific Journal of Public Health 29 (2017): 495–505, 10.1177/1010539517718336. 28679285

[34] A. N. Holowatyj , W. Wen , T. Gibbs , et al., “Racial/Ethnic and Sex Differences in Somatic Cancer Gene Mutations Among Patients With Early‐Onset Colorectal Cancer,” Cancer Discovery 13 (2023): 570–579, 10.1158/2159-8290.CD-22-0764. 36520636 PMC10436779

[35] S. G. Patel , J. J. Karlitz , T. Yen , C. H. Lieu , and C. R. Boland , “The Rising Tide of Early‐Onset Colorectal Cancer: A Comprehensive Review of Epidemiology, Clinical Features, Biology, Risk Factors, Prevention, and Early Detection,” Lancet Gastroenterology & Hepatology 7 (2022): 262–274, 10.1016/S2468-1253(21)00426-X. 35090605

[36] V. V. Tsukanov , A. V. Vasyutin , and J. L. Tonkikh , “Risk Factors, Prevention and Screening of Colorectal Cancer: A Rising Problem,” World Journal of Gastroenterology 31, no. 5 (2025): 98629, 10.3748/wjg.v31.i5.98629. 39926213 PMC11718609

[37] J. Hippisley‐Cox and C. Coupland , “Development and Validation of Risk Prediction Equations to Estimate Survival in Patients With Colorectal Cancer: Cohort Study,” BMJ 357 (2017): j2497, 10.1136/bmj.j2497. 28620089 PMC5471851

[38] Y. Zhou , Z. Lin , S. Xie , et al., “Interplay of Chronic Obstructive Pulmonary Disease and Colorectal Cancer Development: Unravelling the Mediating Role of Fatty Acids Through a Comprehensive Multi‐Omics Analysis,” Journal of Translational Medicine 21 (2023): 587, 10.1186/s12967-023-04278-1. 37658368 PMC10474711

[39] L. Wang , Y. Cai , J. Garssen , P. A. J. Henricks , G. Folkerts , and S. Braber , “The Bidirectional Gut‐Lung Axis in COPD,” American Journal of Respiratory and Critical Care Medicine 207 (2023): 1145–1160, 10.1164/rccm.202206-1066TR. 36883945 PMC10161745

[40] M. D. Mana , A. M. Hussey , C. N. Tzouanas , et al., “High‐Fat Diet‐Activated Fatty Acid Oxidation Mediates Intestinal Stemness and Tumorigenicity,” Cell Reports 35 (2021): 109212, 10.1016/j.celrep.2021.109212. 34107251 PMC8258630

[41] F. S. Yen , M. C. Hou , C. C. J. Wei , et al., “Sodium‐Glucose Cotransporter 2 Inhibitors Use in Patients With Liver Cirrhosis,” Diabetes/Metabolism Research and Reviews 41 (2025): e70070, 10.1002/dmrr.70070. 40696811

[42] F. S. Yen , J. C. Wei , Y. M. Hung , P. Y. Li , C. C. Hsu , and C. M. Hwu , “SGLT2 Inhibitors and Prevention of Cardiovascular Events in Diabetes Patients With and Without Hypertension: A Nationwide Cohort Study,” Journal of General Internal Medicine 41 (2026): 143–152, 10.1007/s11606-025-09847-2. 41055686 PMC12855710

[43] C. H. Chiu , W. Y. Wang , H. Y. Chen , P. L. Liao , G. P. Jong , and T. Y. Yang , “Decreased Risk of Renal Cell Carcinoma in Patients With Type 2 Diabetes Treated With Sodium Glucose Cotransporter‐2 Inhibitors,” Cancer Science 115 (2024): 2059–2066, 10.1111/cas.16157. 38572526 PMC11145143

